# Electron Microscopic Radioautographic Study on Protein Synthesis in Mitochondria of Binucleate Hepatocytes of Aging Mice

**DOI:** 10.1100/tsw.2007.140

**Published:** 2007-06-22

**Authors:** Tetsuji Nagata

**Affiliations:** ^1^ Department of Anatomy and Cell Biology, Shinshu University School of Medicine, Matsumoto, 390-8621, Japan; ^2^ Department of Anatomy, Shinshu Institute of Alternative Medicine, Nagano, 380-0816, Japan

**Keywords:** mitochondria, EM radioautography, protein syntheses, mouse binucleate hepatocytes, aging

## Abstract

In order to study the aging changes of intramitochondrial protein synthesis in mouse hepatocytes, 10 groups of aging mice, each consisting of three individuals, total 30, from fetal day 19 to postnatal year 2, were injected with ^3^H-leucine, a protein precursor, sacrificed 1 h later, and the liver tissues processed for electron microscopic radioautography. On electron microscopic radioautograms obtained from each animal, the numbers of mitochondria, the numbers of labeled mitochondria, and the mitochondrial labeling index labeled with ^3^H-leucine that showed protein synthesis in each hepatocyte, both mononucleate and binucleate cells, were counted and the averages in respective aging groups were compared. From the results, it was demonstrated that the numbers of mitochondria, the numbers of labeled mitochondria, and the labeling indices of intramitochondrial protein syntheses in both mononucleate and binucleate hepatocytes of mice at various ages increased due to development of animals. The numbers of mitochondria, the numbers of labeled mitochondria, and the labeling indices of intramitochondrial protein synthesis in binucleate hepatocytes were more than those of mononucleate hepatocytes at the same aging stages.

## INTRODUCTION

Intramitochondrial nucleic acid syntheses, both DNA and RNA, in mammalian and avian cells were first demonstrated morphologically by the present author by means of electron microscopic radioautography with accurate localization in primary cultured cells of the livers and kidneys of mice and chickens *in vitro*[1,2], and then in some other established cell lines, such as HeLa cells[3,4,5,6] or mitochondrial fractions prepared from *in vivo* cells[7,8,9]. They were later commonly found in various cells and tissues not only *in vitro* obtained from various organs *in vivo*[10,11,12,13,14], but also *in vivo* cells of various organs, such as the salivary glands[15], the liver[16,17,18,19,20,21], the pancreas[22,23], the trachea[24], the kidneys[25], the testis[26,27], the uterus[28,29], the adrenals[30,31], the brains[32], and the retina[33,34,35,36] of mice, rats, and chickens. The protein synthesis in mitochondria of various animal cells has also been extensively studied by both biochemical and morphological procedures[21,37,38,39, 40,41,42,43,44,45,46,47,48,49,50,51]. However, the relationship between the protein synthesis and the aging of individual animals has not yet been clarified. This paper deals with the relationship between the protein synthesis and aging in hepatocytes of mice *in vivo* at various ages by means of electron microscopic radioautography as a part of serial studies on special cytochemistry[47] and radioautographology[48].

## MATERIALS AND METHODS

### The Animals

The liver tissues were obtained from respective animals in 10 small groups of normal ddY strain mice, bred and grown in our laboratory, each consisting of three littermates of both sexes, total 30, aged from embryonic day 19 to postnatal days 1, 3, 9, and 14 and months 1, 2, 6, 12, and 24 (year 2). The embryonic age was based on observation of the vaginal plug of the female mice (vaginal plug = day 0). All the animals were housed under conventional conditions and bred with normal diet (mouse chow Clea EC2, Clea Co., Tokyo, Japan) with access to water *ad libitum*. All the procedures used in this study concerning the animal experiments were in accordance with the guidelines of the animal research committee of Shinshu University School of Medicine, as well as the principles of laboratory animal care (NIH Publication No. 86-23, revised 1985).

### Tissue Processing

Respective animals were injected intraperitoneally with an RI-labeled amino acid, $^3$H-4,5-leucine (Amersham, England, 1002 GBq/m*M*) for protein synthesis 1 h before sacrifices. The dosage of injections was 370 kBq/gm body weight. In case of embryos, $^3$H-4,5-leucine was injected intraperitoneally to the pregnant mother mouse and the embryos were taken out from the uterus after the mother was sacrificed. All the animals were perfused via the left ventricles of the hearts with 0.1 *M* cacodylate-buffered 2.5% glutaraldehyde under Nembutal (Abbott Laboratories, Chicago, IL) anesthesia. The right medial lobes of the livers were excised and three small pieces of the liver tissues (1 × 1 × 1 mm) were immersed in the same fixative at 4˚C for 1 h, followed by postfixation in 1% osmium tetroxide in the same buffer at 4˚C for 1 h, dehydrated in graded series of ethanol and acetone, and embedded in epoxy resin (Epok 812, Oken, Tokyo, Japan).

### Radioautography

Thick sections of 1-µm thickness from respective specimens were cut in sequence on a Porter-Blum MT-2B ultramicrotome (Dupont-Sorvall, Newtown, MA) using glass knives and were collected on glass slides coated with Konica NR-M2 radioautographic emulsion (Konica, Tokyo, Japan) by a dipping method[22,44,48] for light microscopy, exposed for 4 weeks at 4˚C, developed in SDX-1 developer at 20˚C for 5 min, fixed in acid fixer, stained in 0.1% toluidine blue/0.1 *M* sodium phosphate, pH7.4, phosphate, pH7.4, solution. On the other hand, semithin sections of 0.2-µm thickness were cut in sequence on the same ultramicrotome in order to shorten the exposure time for electron microscopic radioautography, collected on collodion-coated copper grid meshes (VECO, Eerbeek, Netherlands) coated with Konica NR-H2 radioautographic emulsion (Konica, Tokyo, Japan) by a wire-loop method[22,42,43,48], exposed for 10 months at 4˚C, developed in phenidon developer at 16˚C for 1 min after 30-sec gold latensification in freshly prepared gold thiocyanate solution, and stained with lead citrate solution for 3 min[22,42,43,48]. The light microscopic radioautograms were examined and photographed with an Olympus Vanox AHB-LB light microscope (Olympus, Tokyo, Japan), while the electron microscopic radioautograms were examined in either a Hitachi H-700 electron microscope (Hitachi, Tokyo, Japan) at accelerating voltages of 200 kV or a JEOL JEM-4000EX electron microscope (JEOL, Tokyo, Japan) at accelerating voltages of 400 kV for observing thick specimens.

### Quantitative Analysis of Electron Micrographs

Twenty electron radioautograms showing cross-sections of mononucleate hepatocytes from each group, based on the electron microscopic photographs taken after observation on 100 hepatocytes at least from respective animals, were analyzed to calculate the number of silver grains by visual grain counting. Because there are functional differences among three regions in the hepatic lobules (central, intermediate and peripheral zones), we observed and collected the data from the cells in the intermediate zone in order to exclude the effects from both portal and central veins.

The number of mitochondria per cell, as well as the number of mitochondria labeled with silver grains showing $^3$H-leucine incorporation, were calculated, respectively. In order to estimate the background fog, the numbers of silver grains in respective radioautograms in 10 circles with the same area size as a mitochondrion selected at random on the plastic sections outside of cell body coated with radioautographic emulsions were counted. The average number of silver grains per mitochondrial area was 0.01–0.03/area in respective groups. Then we considered that almost no chemographic effects of hepatocyte sections, as well as radioautographic procedures, were detected. Therefore, the grain count in each specimen was not corrected. Thus, the mitochondrion that was labeled with more than one silver grain was defined as labeled. From all the data thus obtained, the averages and standard deviations of number of mitochondria, number of labeled mitochondria, and the labeling index of mitochondria in respective aging groups were computed with a personal computer (Macintosh type 8100/100, Apple Computer, Tokyo, Japan). The data were stochastically analyzed using variance and Student's t-test. The differences were considered to be significant at $p < 0.01$.

## RESULTS

### Morphological Observations

By light microscopic observation, the liver tissues from normal ddY strain mice at various ages consisted of hepatocytes, sinusoidal endothelial cells, Kupffer cells, Ito cells, bile ductal epithelial cells, and fibroblasts. The liver tissues were composed mainly of hepatocytes and various hematopoietic cells, while no lobular orientation of hepatocytes was observed at embryonic day 19 and postnatal day 1. At postnatal day 3, orientation of hepatic cells started to form hepatic lobules and the number of hematopoietic cells decreased. At postnatal day 9, hepatic lobules were completely formed and only a few hematopoietic cells remained. From postnatal day 14 to months 1, 2, 6, 12, and 24, hepatocytes and other cell types (such as sinusoidal endothelial cells, Kupffer cells, Ito cells, bile ductal epithelial cells, and fibroblasts) appeared in the same arrangements as completed hepatic lobules. Observing the ultrastructure of hepatocytes by electron microscopy, cell organelles were not well developed at perinatal and early postnatal stages from embryonic day 19 to postnatal day 14. However, these cell organelles appeared well developed at adult and senescent stages from postnatal month 1 to 24 as was previously reported[52].

The binucleate hepatocytes contained two nuclei of equal size, showing that their two nuclei were sectioned at their centers when the specimens were prepared, or two nuclei of unequal size, a large and a small, showing that their nuclei were not sectioned at their centers. On the other hand, there are two types of binucleate cells showing that either the two-paired nuclei are confronting each other with a short distance between or contacting each other without any distance.

## Radioautographic Observations

By observing light microscopic radioautograms, the silver grains were found over both the karyoplasm and cytoplasm of almost all the cells, not only at the perinatal stages from embryonic day 19 to postnatal days 1, 3, 9, and 14, but also at the adult and senescent stages from postnatal month 1 to 2, 6, 12, and 24.

By electron microscopic observation, silver grains were observed in most hepatocytes that contained one nucleus in one cell body at respective aging groups, which were designated as mononucleate hepatocytes. In the mononucleate hepatocytes, silver grains localized not only over euchromatin and nucleoli in the nuclei, but also over many cell organelles, such as endoplasmic reticulum, ribosomes, Golgi apparatus, and mitochondria, as well as cytoplasmic matrices in respective aging groups from perinatal stage at embryonic day 19, postnatal days 1, 3, 9, and 14, to adult and senescent stages at postnatal months 1 to 24, as was formerly reported[52].

On the other hand, some hepatocytes contained two nuclei in one cell body, which was designated as a binucleate hepatocyte. The binucleate hepatocytes were rarely found in the embryos at prenatal embryonic day 19, while they appeared at postnatal stages from postnatal days 1 (Fig. 1A), 3 (Figs. 1B, 1C), 9 (Fig. 1D), and 14 (Fig. 1E) to months 1 (Fig. 1F), 2 (Fig. 2A), 6 (Fig. 2B), 12 (Figs. 2C, 2D), and 24 (Figs. 2E, 2F). The numbers of binucleate hepatocytes increased from newborn stages to juvenile, adult, and senescent stages, as was previously reported[53]. Some of them contained two nuclei of equal size, showing that their two nuclei were sectioned at their centers (Figs. 1B, 1C, 1E, 1F). The two nuclei in Fig. 2C, appearing smaller than Figs. 1B, 1C, 1E, 1F, were cut at a plane off their centers. Other hepatocytes contained two nuclei of unequal size in one cell body, a large and a small, showing that their nuclei were not sectioned at their centers, due to the axes of cutting sections (Figs. 1A, 1D, 2A, 2B, 2D, 2E, 2F). Likewise, the nuclei in Figs. 1A, 1D and 2A, 2B, 2D, 2E, 2F were cut at a diagonal axis in relation to the plane of their nuclei. Some of them contained two nuclei separately (Figs. 1A, 1D, 2A, 2B, 2C, 2D, 2E, 2F), while some of them contained two nuclei in contact with each other (Figs. 1B, 1B, 1C, 1E, 1F). Those binucleate hepatocytes of respective types also showed silver grains localizing over euchromatin and nucleoli in the nuclei as well as many cell organelles, such as endoplasmic reticulum, ribosomes, Golgi apparatus, and mitochondria. As for the intramitochondrial localization of silver grains, they were observed to localize on the mitochondrial membranes, cristae, and matrix (Fig. 3).

## Quantitative Analysis

### *Number of Mitochondria per Cell*

From the results obtained, it was found that almost all the hepatocytes were labeled with silver grains, showing protein synthesis in their nuclei, cytoplasm, and mitochondria. Preliminary quantitative analysis on the number of mitochondria in 10 mononucleate hepatocytes whose nuclei were intensely labeled with many silver grains (more than 10 per nucleus) and other 10 mononucleate hepatocytes whose nuclei were not so intensely labeled (number of silver grains less than nine) in each aging group revealed that there was no significant difference between the number of mitochondria, number of labeled mitochondria, and the labeling indices in both types of hepatocytes[52]. Thus, the numbers of mitochondria, the numbers of labeled mitochondria, and the labeling indices were calculated in 10 binucleate hepatocytes selected at random in each animal in respective aging stages, regardless whether their nuclei were very intensely labeled or not, except the prenatal stage at embryonic day 19, because no binucleate cell was found at this stage. The results obtained from the numbers of mitochondria in binucleate hepatocytes showed an increase from the postnatal day 1 (66.2/cell), to 3 (66.4/cell) and 14 (81.8/cell), to postnatal months 1 (89.9/cell), 2 (95.1/cell), and 6 (102.1/cell), reaching the maximum at month 12 (128.0/cell), then a decrease to year 2 (93.9/cell) as shown in Fig. 4. The increase and decrease were stochastically significant ($p < 0.01$).

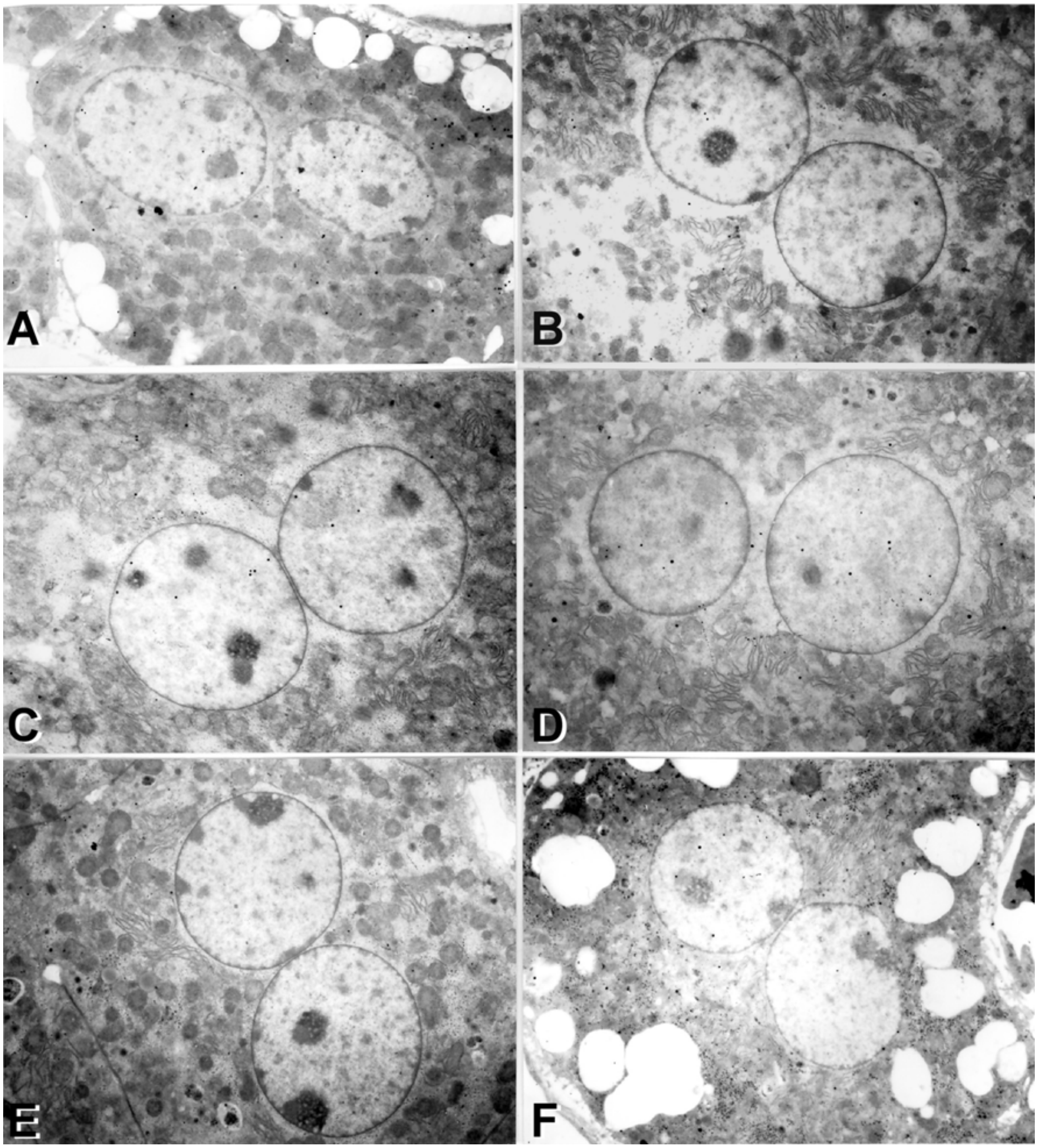

**FIGURE 1**. Electron microscopic radioautograms of binucleate hepatocytes of postnatal newborn, juvenile, and young mice, labeled with $^{3}$H-leucine, showing protein synthesis. (A) Newborn mouse aged at postnatal day 1. Note that several silver grains are observable over the mitochondrial membranes and crista of several mitochondria at right. ×5,000. (B) Suckling mouse aged at postnatal day 3. Note that a few silver grains are observable over the mitochondrial membranes and crista of a few mitochondria at right bottom. ×5,000. (C) Weanling mouse aged at postnatal day 9. Note that the paired nuclei are contacting each other and a few silver grains are observable over a few mitochondria at top left. ×5,000. (D) Weanling mouse aged at postnatal day 9. Note that the paired nuclei are separated and a few silver grains are observable over a few mitochondria at top and bottom left. ×3,000. (E) Juvenile mouse aged at postnatal day 14. Note that the paired nuclei are contacting each other and a few silver grains are observable over a few mitochondria at bottom left and right. ×5,000. (F) Young mouse aged at postnatal day 30. Note that a few silver grains are observable over a few mitochondria at bottom left. An endothelial cell can be seen at right. ×5,000.

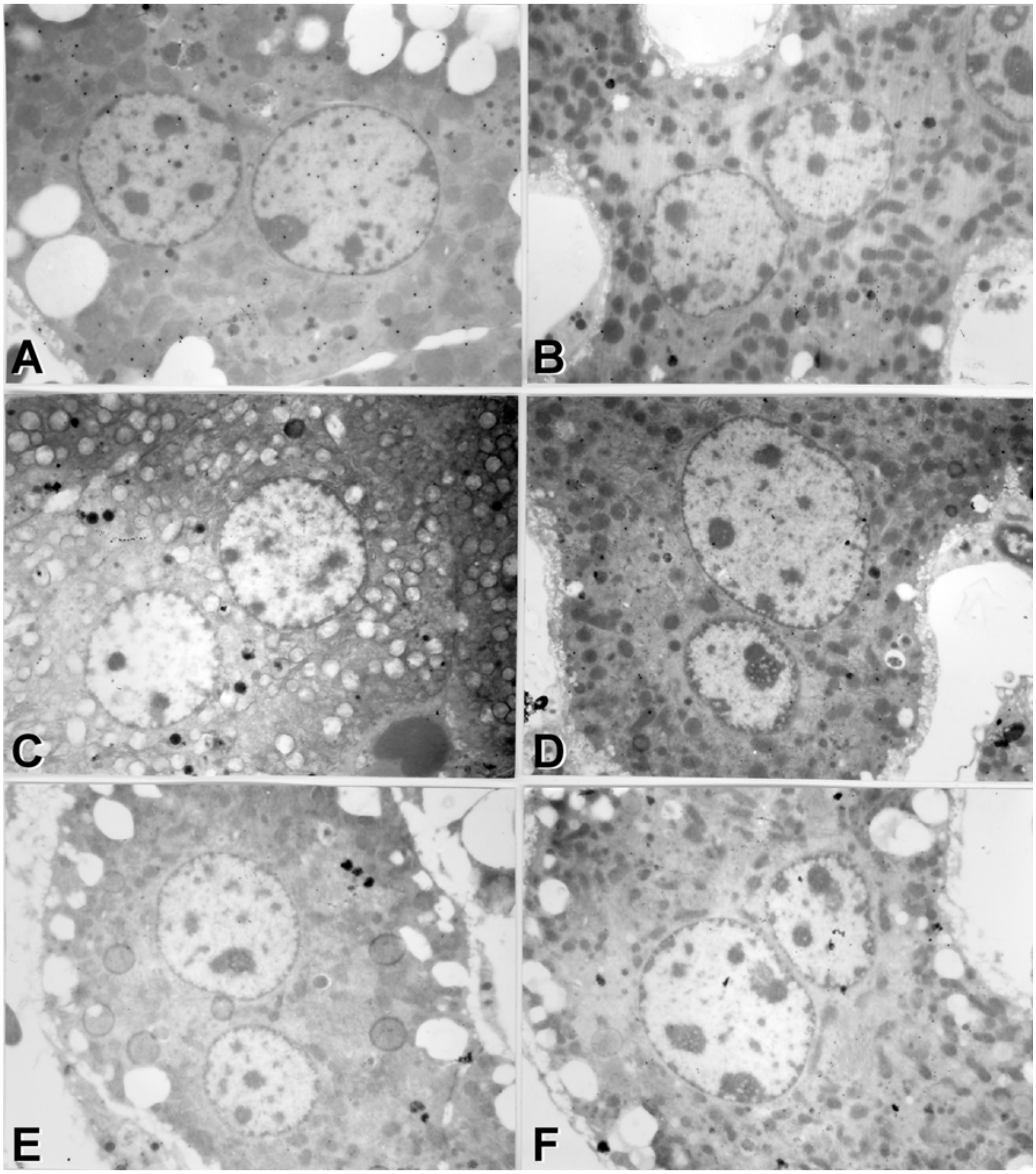

**FIGURE 2**. Electron microscopic radioautograms of binucleate hepatocytes of adult mice aged at postnatal months 2 to 24, labeled with $^3$H-leucine, showing protein synthesis. (A) Adult mouse aged at postnatal month 2. Many silver grains due to $^3$H-leucine can be seen in the two nuclei, especially over the euchromatin as well as over many mitochondria throughout the cytoplasm. ×5,000. (B) Old adult mouse aged at postnatal month 6. Note that the paired nuclei are separated and several silver grains are observable over several mitochondria. ×5,000. (C) Senile mouse aged at postnatal month 12 (1 year). Only a few silver grains can be seen localizing over mitochondrial membranes of the three mitochondria at top right and left. ×5,000. (D) Senile mouse aged at postnatal month 12 (1 year). Note that several silver grains can be seen over several mitochondria at bottom right and left. ×5,000. (E) Senile mouse aged at postnatal month 24 (2 years). Note that no silver grain can be observed over any mitochondria. ×5,000. (F) Senescent mouse aged at postnatal month 24 (2 years). Note that only a few silver grains are observable over a few mitochondria at bottom right. Less silver grains can be seen as compared with the younger animals. ×5,000.

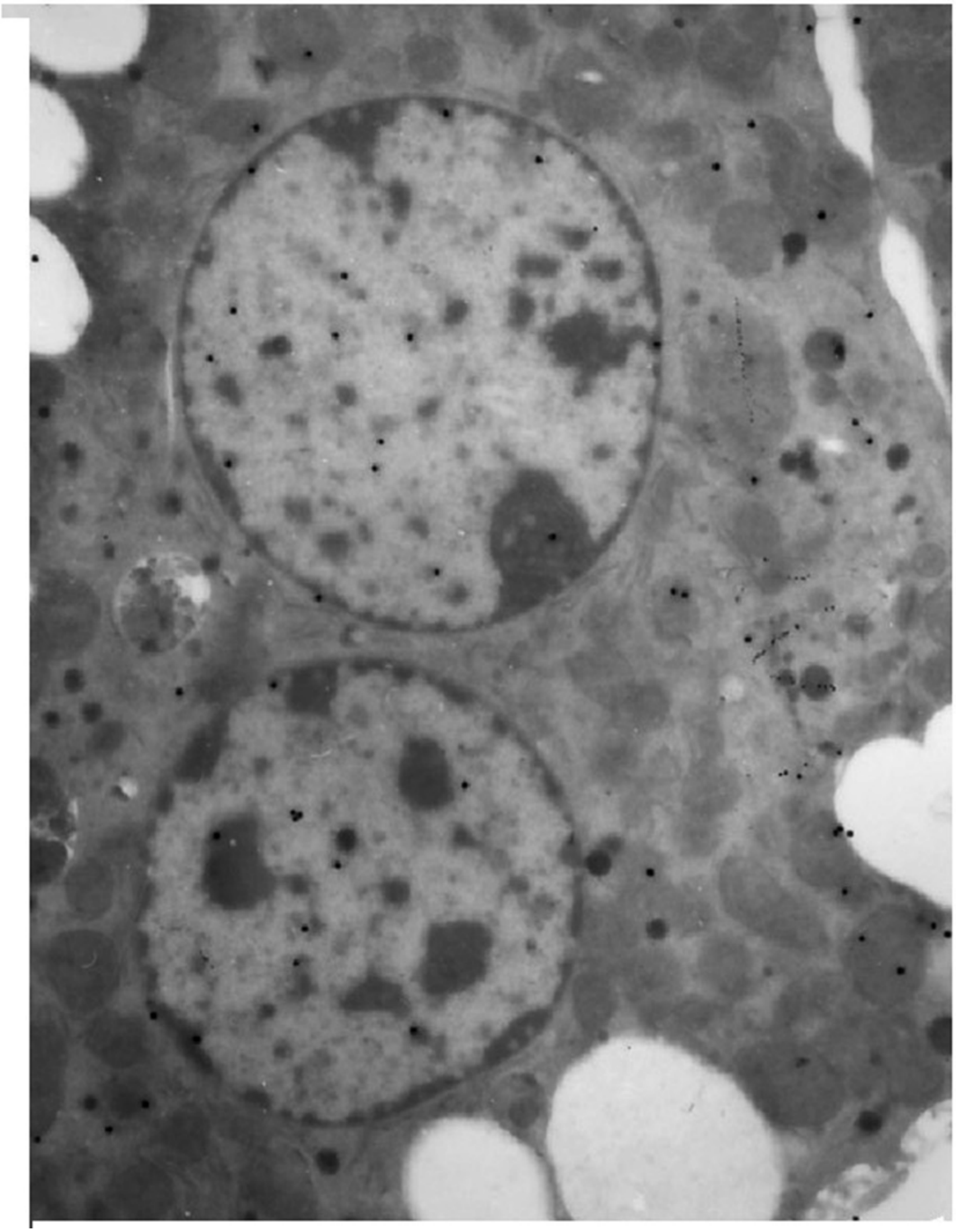

**FIGURE 3**. High-power magnification electron microscopic radioautogram of a binucleate hepatocyte of an adult mouse aged at postnatal month 2, labeled with $^{3}$H-leucine, showing protein synthesis. Many silver grains due to $^{3}$H-leucine can be seen in the two nuclei, especially over the euchromatin as well as over many mitochondria throughout the cytoplasm. ×20,000.

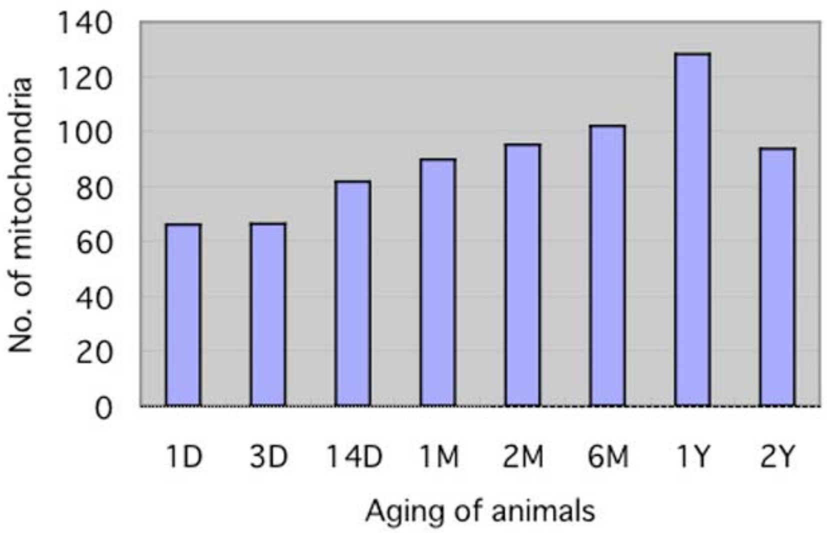

**FIGURE 4**. Histogram demonstrating aging change of the total number of mitochondria per cell profile area in binucleate hepatocytes labeled with $^{3}$H-leucine at respective aging groups from postnatal day 1 to postnatal month 24.

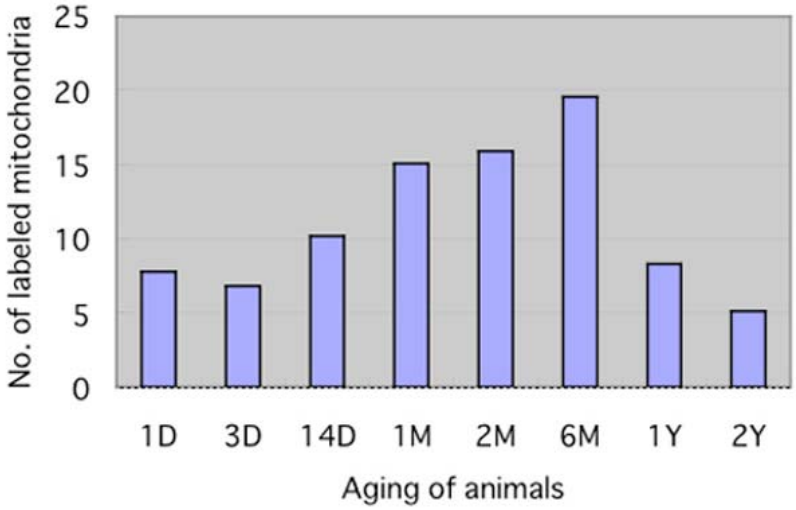

**FIGURE 5**. Histogram demonstrating aging change of the total number of mitochondria labeled with $^{3}$H-leucine, showing protein synthesis per cell profile area in binucleate hepatocytes, at respective aging groups from postnatal day 1 to postnatal month 24.

## *Mitochondrial Protein Synthesis*

The results obtained from visual counting of the numbers of mitochondria labeled with silver grains from 10 mononucleate hepatocytes of each animal labeled with $^{3}$H-leucine, demonstrating protein synthesis in 10 aging groups at postnatal days 1, 3, and 14, months 1 and 6, and years 1 and 2, are plotted in Fig. 5. The labeling indices in respective aging stages were calculated from the numbers of labeled mitochondria and the numbers of total mitochondria per cell, which were plotted in Fig. 6. The results showed that the numbers of labeled mitochondria with $^{3}$H-leucine, showing protein synthesis, increased from postnatal day 1 (7.3/cell) to day 3 (6.8/cell) and 14 (10.2/cell), and months 1 (15.0/cell) and 2 (15.9/cell), reached the maximum at month 6 (19.6/cell), then decreased to years 1 (8.3/cell) and 2 (5.1/cell). On the other hand, the

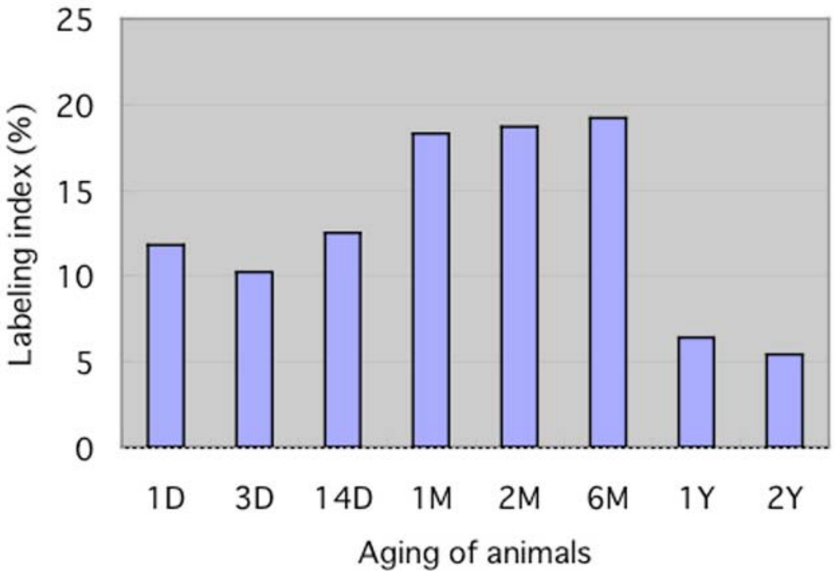

**FIGURE 6**. Histogram demonstrating aging change of the labeling index of mitochondria in binucleate hepatocytes labeled with $^3$H-leucine, showing protein synthesis (number of labeled mitochondria/number of total mitochondria), at respective aging groups from postnatal day 1 to postnatal month 24.

labeling indices increased from postnatal day 1 (11.8%) to 3 (10.2%) and 14 (12.5%), months 1 (18.3%) and 2 (18.7%), reached the maximum at month 6 (19.2/cell), then decreased to years 1 (6.4%) and 2 (5.5%). Stochastical analysis revealed that the increases and decreases of the numbers of labeled mitochondria, as well as the labeling indices from the newborn stage to the adult and senescent stages, were significant ($p < 0.01$).

## DISCUSSION

With regard to the macromolecular synthesis in various cells in various organs of experimental animals as observed by light and electron microscopic radioautography, it is well known that the silver grains demonstrate DNA, RNA, and protein syntheses due to the radiolabeled precursors, such as $^3$H-thymidine, $^3$H-uridine, and $^3$H-leucine, respectively[1,2,3,22,43,44,48]. The present results obtained from the livers of aging mice revealed that the incorporation of $^3$H-leucine, indicating protein synthesis, resulted in silver grain localization over the nuclei and cell bodies of almost all binucleate hepatocytes from newborn postnatal days 1, 3, and 14 to adult and senescent stages at postnatal months 1, 2, 6, 12, and 24, which showed the localization of newly synthesized proteins in the nuclei and the cytoplasmic cell organelles including mitochondria. From the results obtained at present, the numbers of mitochondria in respective binucleate hepatocytes increased from postnatal day 1 (66.2/cell), reached the maximum (128.2/cell) at postnatal year 1, and decreased to year 2 (93.8/cell) due to aging, while the numbers of labeled mitochondria with silver grains due to $^3$H-leucine incorporation, demonstrating intramitochondrial protein synthesis, also increased from postnatal day 1 (7.3/cell), reached the maximum (19.6/cell) at postnatal month 6, and decreased to year 2 (5.1/cell). The labeling indices of mitochondria labeled with $^3$H-leucine also showed an increase from postnatal day 1 (11.8%), reached the maximum (19.2%) at postnatal month 6, and decreased to year 2 (5.5%). We previously studied the intramitochondrial protein synthesis in the livers of aging mice from prenatal embryonic day 19 to postnatal days 1, 3, 9, and 14, to adult and senescent stages at postnatal months 1, 2, 6, 12, and 24, which showed the localization of newly synthesized proteins in the nuclei and the cytoplasmic cell organelles including mitochondria of almost all hepatocytes[52]. From the results obtained in the previous study, the numbers of mitochondria in respective mononucleate hepatocytes increased from embryonic day 19 (34.5/cell), to postnatal stages reaching the maximum (60.7/cell) at postnatal month 6, and decreased to year 2 (51.2/cell), while the numbers of labeled mitochondria with silver grains due to $^3$H-leucine incorporation, demonstrating intramitochondrial protein synthesis, also increased from embryo day 19 (8.3/cell), to postnatal stages reaching the maximum (11.2/cell) at postnatal month 1, then decreased to postnatal year 2 (2.3/cell). The labeling indices of mitochondria labeled with $^3$H-leucine increased from embryonic day 19 (20.1%), to postnatal stages reaching the maximum (23.1%) at postnatal day 14, then decreased to postnatal year 2 (4.4%). Comparing these results from mononucleate cells with the present results from binucleate cells, it is obvious that the numbers of mitochondria and the numbers of labeled mitochondria were more in the binucleate hepatocytes than the mononucleate, while the labeling indices in mononucleate hepatocytes were higher than binucleate. It is also interesting to find that the timing of the maximal values of the numbers of mitochondria, numbers of labeled mitochondria, and the labeling indices in mononucleate cells appeared earlier than in binucleate cells. These results indicate that binucleate cells contain more mitochondria than mononucleate cells at respective aging groups and more mitochondria synthesize protein in each binucleate cell at various ages. However, the percentage of mitochondria synthesizing proteins was lower in the binucleate than in the mononucleate.

Concerning the protein synthesis in mitochondria in animal cells or plastids in plant cells, it has been observed in various cells of some plants[53,54,55], several kinds of animals (such as catfish[56], hamster[57] and rat[58]), or human patients[59,60,61], by means of various approaches. Most of these authors observed mitochondrial protein synthesis in various cells by biochemical procedures. However, no evidence showing protein synthesis in mitochondria or plastids of any kind of cells, either plants or animals, has been demonstrated by morphological methods, such as light or electron microscopy, except in only a few papers published from our laboratory[1,46,50,62,63,64,65,66]. Among these studies, we first demonstrated that the mitochondria of cultured cells could incorporate $^3$H-leucine and synthesized proteins *in vitro*[1]. Then, we demonstrated that the numbers of mitochondria and the numbers of labeled mitochondria incorporating $^3$H-leucine in mouse mononucleate hepatocytes *in vivo* increased from prenatal stage to postnatal adult stage at month 1, but the labeling indices did not show any significant increase[46,50,62,63,64,65,66]. In the present study, it was clearly demonstrated that the numbers of mitochondria, the numbers of labeled mitochondria with $^3$H-leucine, and the labeling indices of mouse binucleate hepatocytes showed significant increases and decreases from perinatal stage to adult and senescent stages. The present study showing the silver grain localization in the mitochondria of mouse binucleate hepatocytes incorporating $^3$H-leucine, as observed by electron microscopic radioautography, should be the first report demonstrating the protein synthesis in the mitochondria of binucleate cells in connection to the animal aging from perinatal stages to postnatal adult and senescent stages as studied systematically. It should be worthy of notice that the silver grains localized over the mitochondrial membranes, cristae, and matrices of all hepatocytes at various ages. The results indicated that the protein synthesis as shown by $^3$H-leucine incorporations demonstrated the synthesis of structural proteins in the mitochondrial membranes, cristae, and matrices.

As for the macromolecular synthesis in various cells in various organs of experimental animals observed by electron microscopic radioautography, it is well known that the silver grains demonstrate DNA synthesis due to radiolabeled $^3$H-thymidine, while $^3$H-uridine demonstrates RNA synthesis and $^3$H-leucine demonstrates protein synthesis in both karyoplams and cytoplasm[1,2,3,4,5,8,9,12,13, 22,43,44,48,67]. The previous studies obtained from the livers of aging mice revealed that silver grains incorporating either $^3$H-thymidine or $^3$H-uridine were observed not only over the nuclei of some hepatocytes, but also over the mitochondria, showing intramitochondrial DNA and RNA synthesis[65,66]. When the labeling time of cultured cells incubated in the media containing RI-labeled precursors *in vitro* or when the sacrifice time of the experimental animals were extended after the administrations of RI-labeled compounds *in vivo*, the numbers of silver grains in respective cell compartments of various cells in radioautograms increased according to the time course[1,2,41,42,43,44,45,47,48,50,53,64,65,66,67]. Therefore, we employed only one time point at 1 h after RI-injections in order to compare the densities of synthesized proteins in hepatocyte mitochondria excluding the time course in the present experiment.

With regard to the incorporation of $^{3}$H-thymidine into mitochondria demonstrating DNA synthesis, many authors previously reported that it was observed by means of electron microscopic radioautography in lower organisms, such as slime mold[68,69], tetrahymena[70], or chicken fibroblasts in tissue culture under abnormal conditions[71,72]. Most of these authors, however, used old-fashioned developers consisting of methol and hydroquinone (MQ developer), which produced coarse spiral silver grains resulting in inaccurate localization over cell organelles, especially mitochondria, when observed by electron microscopy. Thus, most of these authors, except Nagata[3,4,5,6,7,67], showed photographs of electron radioautographs with large spiral-formed silver grains (2–3 μm in diameter) localizing not only over the mitochondria, but also outside the mitochondria. In order to obtain smaller silver grains, we first used elon-ascorbic acid developer after gold latensification[3,4,7,67], which produced comma-shaped smaller silver grains (0.3–0.8 μm in diameter), better than spiral silver grains (2–3 μm in diameter), then later we used phenidon developer after gold latensification, producing dot-like smaller silver grains (0.2–0.3 μm in diameter) localizing only inside the mitochondria showing ultrahigh resolution of radioautograms[22,43,44,48] as shown in Figs. 1 and 2 in this study. Thus, these papers from our laboratory were the first that demonstrated intramitochondrial DNA, RNA, and protein syntheses incorporating $^{3}$H-thymidine, $^{3}$H-uridine, and $^{3}$H-leucine, respectively, with accurate intramitochondrial localization in avian and mammalian cells[1,2,3,4,5,6,7,67]. Concerning the resolution of electron microscopic radioautography, on the other hand, several authors discussed the sizes of silver grains under various experimental conditions and calculated various values of resolutions[3,4,73,74,75]. Those authors who used the MQ developers maintained the resolution to be 100–160 nm[73,74,75], while those authors who used the elon-ascorbic acid developer[5,6,76] calculated it to be 25–50 nm. When we used phenidon developer at 16˚C for 1 min after gold latensification, we could produce very fine dot-shaped silver grains and obtained the resolution around 25 nm[22,43,44,77]. For the purpose of analyzing electron radioautographs, Salpeter et al.[74] suggested to use the half-distance and proposed to use very complicated calculations through which respective coarse spiral-shaped silver grains were judged to be attributable to the radioactive source in a certain territory within a resolution boundary circle. However, since we used phenidon developer after gold latensification to produce very fine dot-shaped silver grains, we judged only the silver grains that were located in the mitochondria that were very fine dot-shaped ones to be attributable to the mitochondria without any problem with the resolution around 25 nm as was formerly discussed[5,6]. As for the section thickness, we used thicker semithin sections at 0.2-μm thickness, which did not affect the HD value of this experiment since we used tritium as RI, which emitted beta rays with very low energy having very short range at 0.2 μm in the emulsions. Thus, the numbers of labeled mitochondria as well as the labeling indices that were calculated from the results obtained from the numbers of mitochondria over which the silver grains really existed without adding any hypothetical silver grains which should be less than 10% (only several %) if added under the experimental conditions that we carried out in this experiment were not corrected.

On the other hand, the incorporation of $^{3}$H-thymidine into mitochondria demonstrating DNA synthesis were formerly observed by means of electron microscopic radioautography not only in lower organisms, such as slime mold[68,69] and tetrahymena[70], but also in higher animals, such as chicken fibroblasts in tissue culture under abnormal conditions[71]. Then we also demonstrated intramitochondrial DNA synthesis incorporating $^{3}$H-thymidine or RNA synthesis incorporating $^{3}$H-uridine in some other established cell lines originated from human being such as HeLa cells[3,4,5,6] or mitochondrial fractions prepared from *in vivo* mammalian cells, such as rat and mouse livers[7,8,9]. It was later commonly found in various cells and tissues *in vitro* obtained from various organs, such as the cultured human uterus cancer cells HeLa[13,78], cultured rat sarcoma cells[12], mouse liver and pancreas cells *in vitro*[11,14], but also *in vivo* cells obtained from various organs, such as the salivary gland[15], the liver[16,17,18,21], the pancreas[22,23], the trachea[24], the kidney[25], the testis[26,27], the uterus[28,29,79,80], the adrenal gland[30,31], the brain[32], and the eye[33,34,35,36,37] of mice, rats, and chickens. Thus, it is clear that all the cells in various organs of various animals synthesize DNA and RNA not only in their nuclei, but also in their mitochondria.

The relationship between the cell cycle and the intramitochondrial DNA as well as RNA syntheses was formerly studied in cultured synchronized cells and it was clarified that both the intramitochondrial DNA and RNA syntheses were performed without any nuclear involvement[4]. However, the relationship between the aging of individual animals and the DNA and RNA syntheses has not yet been clarified except in a few papers first published by Korr and associates on mouse brain[81,82,83,84]. They reported both nuclear DNA repair, measured as nuclear unscheduled DNA synthesis, and cytoplasmic DNA synthesis labeled with $^{3}$H-thymidine in several types of cells in brains, such as pyramidal cells, Purkinje cells, granular cells, glial cells, endothelial cells, ependymal cells, and epithelial cells as observed by light microscopic radioautography using paraffin sections. They observed silver grains over cytoplasm of these cells by light microscopy and maintained that it was reasonable to interpret these labeling as $^{3}$H-DNA outside the nuclei, which theoretically belonged to mitochondrial DNA without observing the mitochondria by electron microscopy. From the results, they concluded that distinct types of neuronal cells showed a decline of both unscheduled DNA and mitochondrial DNA syntheses with age in contrast that other cell types, glial and endothelial cells, did not show such age-related changes without counting the number of mitochondria in respective cells neither counting the number of labeled mitochondria nor calculating the labeling indices of mitochondria at respective aging stages. Thus, their results from the statistics obtained from the cytoplasmic grain counting seem to be not accurate without observing mitochondria directly by electron microscopy.

To the contrary, we first showed the relationship between the DNA synthesis and aging in hepatocytes of mice *in vivo* at various ages from prenatal embryo to postnatal senescent adult at 2 years by means of electron microscopic radioautography observing the small dot-like silver grains, due to incorporations of $^{3}$H-thymidine, which were developed with phenidon developer after gold latensification exactly localized inside the mitochondria[65]. We demonstrated that increases and decreases were observed in the numbers of mitochondria per profile area of hepatocyte cytoplasm and the numbers of labeled mitochondria as well as labeling indices of DNA synthesis showing $^{3}$H-thymidine incorporations by direct observation on mitochondria at electron microscopic level. Likewise, we also demonstrated that increases and decreases were observed by direct observation on hepatocyte mitochondria at electron microscopic level and obtaining accurate mitochondrial numbers and labeling indices labeled with $^{3}$H-uridine, demonstrating RNA synthesis[66], as well as protein synthesis labeled with $^{3}$H-leucine[52]. However, the difference of protein synthesis between mononucleate and binucleate hepatocytes has not been elucidated. Thus, this paper should be the first to show the relationship between the protein synthesis and aging in mononucleate and binucleate hepatocytes of mice *in vivo* at various ages from prenatal to postnatal juvenile, adult, and senescent stages by means of electron microscopic radioautography observing the small dot-like silver grains, due to incorporations of $^{3}$H-leucine, which exactly localized inside the mitochondria.

It is well known that considerable numbers of binucleate hepatocytes appear in adult and senescent stages[50,63,65,66]. We formerly observed that the quantity of DNA and RNA synthesis as expressed by grain counting in the karyoplasm and cytoplasm of both mononucleate and binucleate cells in mouse hepatocytes showed increases and decreases reaching the maxima at postnatal month 2 in the case of mononucleate cells and at month 6 in the case of binucleate cells[50,63,65,66]. It was noted that the differences of grain counts between the mononucleate and binucleate cells in the same aging groups were stochastically significant. These results indicated that the amount of DNA and RNA synthesized and distributed in the karyoplasm and cytoplasm of each binucleate cell was much more than each mononucleate cell in respective aging groups[50,63,65,66]. Considering these results, the differences between the mitochondrial protein synthesis of mononucleate and binucleate hepatocytes were also demonstrated in the present study as was observed in DNA and RNA syntheses. The results obtained from the liver at present should form a part of special radioautographology[48], i.e., application of radioautography to the liver, as well as a part of special cytochemistry[47], as was recently reviewed by the present author. We expect that such special radioautographology and special cytochemistry should be further developed in all the organs in the future.

From the results obtained at present, it was concluded that almost all the hepatocytes of mice at various ages, from prenatal embryos to postnatal newborn, young, adult, and senescent animals, were labeled with silver grains, showing protein synthesis with $^3$H-leucine in their mitochondria. Quantitative analysis on the numbers of mitochondria in binucleate hepatocytes resulted in an increase from the perinatal days to postnatal months 1, 2, and 6, reached the maximum at postnatal year 1, then decreased to year 2, in contrast to the mitochondria in mononucleate hepatocytes that were less than binucleate hepatocytes, and reached the maximum at postnatal month 6. The numbers of labeled mitochondria with $^3$H-leucine, showing protein synthesis in binucleate hepatocytes, increased from perinatal days to postnatal months, reached the maximum at postnatal month 6, then decreased to year 2, in contrast to the numbers in mononucleate hepatocytes that were less than binucleate hepatocytes, and reached the maximum at postnatal month 1. The labeling indices of mitochondria in binucleate hepatocytes increased from perinatal stage to postnatal months, reached the maximum at postnatal month 6, then decreased to year 2, while the labeling indices in mononucleate hepatocytes were higher than those in binucleate hepatocytes and reached the maximum at postnatal day 14. These results demonstrated the different patterns of aging changes of protein synthesis in the mitochondria of mononucleate and binucleate hepatocytes.

## ACKNOWLEDGMENTS

This study was supported in part by a Grant-in-Aids for Scientific Research from the Ministry of Education, Science and Culture of Japan (No. 02454564) while the author worked at Shinshu University School of Medicine, as well as a Grant for Promotion of Characteristic Research and Education from the Japan Foundation for Promotion of Private Schools (1997, 1998 1999, 2000) while the author worked at Nagano Women's Jr. College. The author is also grateful to a Grant-in-Aids for Scientific Research from the Japan Society for Promotion of Sciences (No. 18924034) while the author has been working at Shinshu Institute of Alternative Medicine since 2005. The author thanks Dr. Kiyokazu Kametani, Technical Official, Research Center for Instrumental Analysis, Shinshu University, for his technical assistance during the course of this study.
